# Oscillations in Rational Economies

**DOI:** 10.1371/journal.pone.0087820

**Published:** 2014-02-05

**Authors:** Yuriy Mishchenko

**Affiliations:** Toros University, Mersin, Turkey; Universidad Veracruzana, Mexico

## Abstract

Economic (business) cycles are some of the most noted features of market economies, also ranked among the most serious of economic problems. Despite long historical persistence, the nature and the origin of business cycles remain controversial. In this paper we investigate the problem of the nature of business cycles from the positions of the market systems viewed as complex systems of many interacting market agents. We show that the development of cyclic instabilities in these settings can be traced down to just two fundamental factors – the competition of market agents for market shares in the settings of an open market, and the depression of market caused by accumulation of durable overproduced commodities on the market. These findings present the problem of business cycles in a new light as a systemic property of efficient market systems emerging directly from the free market competition itself, and existing in market economies at a very fundamental level.

## Introduction

Economic or business cycles are some of the most noted features of market economies, spanning historically over 200 years and ranking among the most serious of economic problems [1], [2]. Despite a number of economic theories proposed to explain the nature of economic cycles, including the theories of multiplier-accelerator [3], inventory cycles [2], [4], politically based cycles [5], [6], credit/debt cycles [7], [8], the real business cycles [9]–[12], and many others [2], [13]–[16], the nature of such cycles remains highly controversial. Notably, most existing economic theories associate economic cycles with various economically suboptimal and irrational behaviors such as speculative and crowd effects [17], inefficiencies in business decision making [4], exogenous shocks such as new technologies, political crises and wars [10]–[12], political interventions [5], [6], etc. However, the dramatic persistence of economic cycles throughout the 200 years of recorded economic history leads one to question the soundness of such views.

In mainstream economic theory, business cycles are associated with the fluctuations in aggregate demand coupled with so called accelerator and multiplier effects [1], [18]–[21]. Accelerator effect is the tendency of businesses to increase their investment spendings beyond usual levels in growing economies and to lower that in shrinking economies. Multiplier effect is the tendency of increased investment spendings to additionally stimulate economy as the result of the money turnover. The multiplier-accelerator model, if represented in a mathematical form [3], [22], does give rise to oscillatory patterns in the fluctuations of aggregate demand; however, for that it relies on economically “irrational” tendency of businesses to continue expanding their investments even in already oversaturated but still growing economy, as embodied by the accelerator effect, and leaves without explanation the nature of the initial fluctuation that gives rise to subsequent oscillations. A completely different perspective on business cycles have been assumed by the more recent real business cycles theory [9]–[12]. The real business cycles theory supposes that business cycles always have an exogenous cause such as disruptive new technologies, geo-economical changes, political crises, wars, etc. and, in that sense, are just a response to the changes in real markets’ conditions. Credit/debt cycles theory [7], [8], on the other hand, attributes business cycles to the dynamics of over-borrowing by businesses during the times of economic booms, followed by economic slowdown and, finally, a debt crisis and a recession. Political cycles theory [5], [6] attributes business cycles directly to political manipulations and improper government interventions. Some of the oldest views on business cycles in Marxian economics [13], [14], [23] associate business cycles with the intrinsic property of businesses to lose profitability and fail with time, translating into recessions accompanied by mass unemployment, wealth inequality and economical restructuring aimed at recovering profitability.

In recent years a number of works, especially in the context of the new physics of complex systems, had emerged pursuing the understanding of market phenomena from the perspective of market systems viewed as complex systems of interacting agents [24]–[33]. Such works had offered new insights into phenomena such as financial fluctuations [24], [26], [34]–[38], market panics [29], [39]–[41], financial contagion [42]–[45], and many others. In this work, we present new findings for the problem of business cycles assuming a similar perspective on the business cycles as a systemic property of market systems originating from the collective behavior of rational market agents. We show that the development of business cycles in such settings can be traced down to just two factors – systemic overproduction caused by the competition of rational market agents for market shares in the settings of an open market economy and the depression of the market caused by sustained accumulation of thus overproduced durable commodities.

Subsequently, we focus on an example of extremely basic and fundamental economic model of a single commodity market with several competitive producers. We show that this economic setting is characterized by the property known otherwise as the “Tragedy of the commons” [46]. The tragedy of the commons is an instance of a public goods dilemma that arises when several agents are allowed to collectively exploit a shared resource. It is known that in such settings the individually optimal decisions of the agents can lead to collectively disastrous outcomes in the form of the resource’s overexploitation and even its complete destruction [47]. For open markets, we show here that the markets themselves can be viewed as such a common “resource” being “exploited” by producers, and that the “overexploitation” of this resource in the circumstances similar to that of the tragedy of the commons manifests itself as overproduction crises. Such overproduction coupled with the depressing effect on the market of the accumulation of overproduced durable commodities can cause the market to crash and initiate an economic cycle.

**Figure 1:**
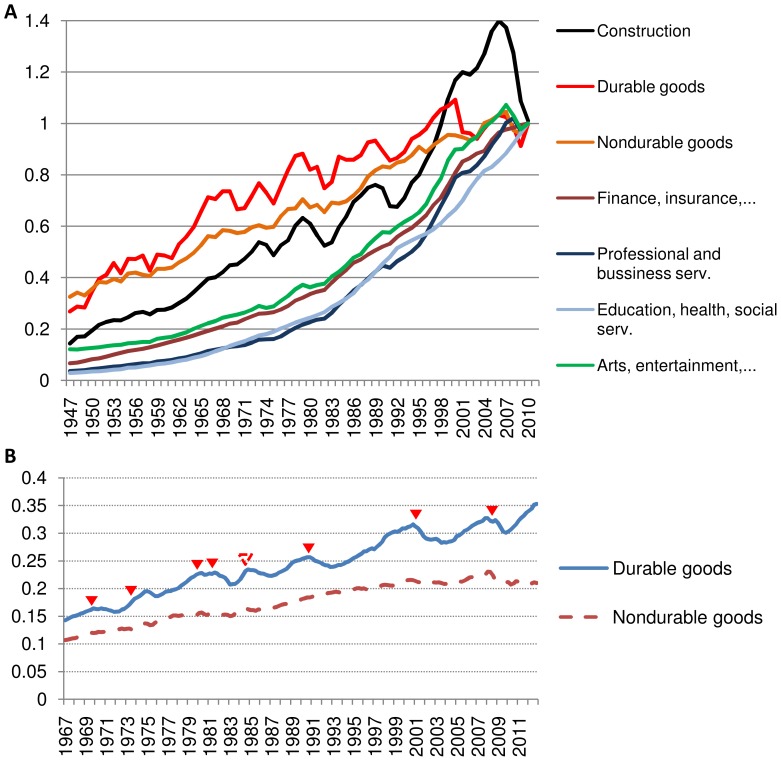
Business cycles appear prominently throughout economic history and display certain prominent patterns. A) Business cycles are known to affect primarily durable goods manufacturing and construction, while nondurable goods and services remain essentially unaffected. Graph A shows the value added by different industries in the U.S. economy since 1947, normalized to the year 2010. The differences between durable goods and construction and nondurable goods and services are clearly visible. B) Business cycle exhibits the pattern of inventories accumulation prior to and reduction during the recession part of the cycle. Graph B shows the changes in durable and nondurable goods inventories in the U.S. economy (in trillions of chained 2005 US dollars) during the last 7 recessions. The beginning of each recession is marked with a triangle. The pattern is clearly visible in durable goods but not nondurable goods inventories. Dashed triangle shows one case of the pattern appearing without an official recession. (Source: U.S. Bureau for Economic Analysis).

The development of economic cycles is thus linked to the free market competition and the ability of overproduced commodity to accumulate on the market, that is, we observe that the cycles develop in the markets of durable goods but do not appear in the markets of nondurable goods. Interestingly, this is otherwise a well-known property of real economic cycles [21], [48]. For instance, in Fig. 1A we show the U.S. economic output by industries in 1947–2010 (U.S. Bureau for Economic Analysis). While the business cycles affect profoundly the durable goods manufacturing and construction, the nondurable goods and services remain practically unaffected by the business cycles throughout the entire period. Our model is also found to produce characteristic patterns in the evolution of commodity’s inventories, with an excess accumulation of inventories immediately prior to and drop during and after the recession segment of the cycle. Indeed, such pattern is also present in real economies. For instance, in Fig. 1B we graph durable and nondurable goods inventories in the U.S. economy in 1967–2010 (U.S. Bureau for Economic Analysis), with special attention to the last 7 recessions. The pattern of inventories’ over-accumulation immediately prior to the recessions and drop during the recessions is clearly visible in the durable goods inventories. In fact, co-cyclic pattern of inventories in business cycles is a well-known feature of real economies [21], [48].

The present findings, therefore, cast the problem of economic cycles in a new light as an emergent property of efficient market systems originating directly from the free competition in the settings of open markets, and inherent to open market systems at a very fundamental level.

## Materials and Methods

### Systemic Overproduction Crises in Open Market Economies

We consider a model of a single commodity market with several fully informed and rational competitive producers. In the model, each producer chooses the amount of the commodity 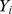 that she wants to produce, while the demand *X* is assumed to be a constant. The producers choose the production levels individually and rationally so as to maximize individual profits defined as,
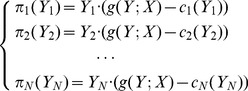
(1)


Here, 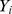 is the production of the *i* ^th^ producer (*i = *1,2,…,*N*), 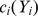 is the production cost of the *i* ^th^ producer, and 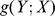 is the market’s return. The return function 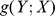 depends on the total production output 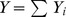 and the market size *X*, and is a non-increasing function of *Y* and a non-decreasing function of *X*, following the standard supply-demand arguments [21]. For simplicity, we shall assume here that all producers are equal, that is 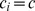 for all *i*.

The tragedy of the commons is a public goods dilemma in which a group of players is allowed to exploit a common resource (a “commons”), commonly exemplified by a shared pasture, fishery, or forest [46]. Each player is free to choose a level of the resource exploitation (for example, the number of cattle to put on the pasture etc.) and does so independently and rationally according to one’s self-interest. The payoff of each decision is given by an equation identical to Eq. (1), in which 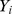 is understood as the level of the resource exploitation by player *i* and 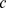 is the associated cost. An essential property of the tragedy of the commons is that the resource’s return function, 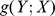, is decreasing with exploitation *Y*; this is a typical situation for most shared resources [47]. Given that assumption, it can be shown that the Nash equilibrium of the players in this situation causes the resource to be necessarily overexploited [49]–[52].

Briefly, the Nash equilibrium in a non-cooperative game is defined as such an equilibrium point 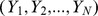 in which none of the players can further increase their payoffs by any unilateral action [52]. Here, such unilateral actions correspond to increase or decrease of 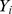; therefore, this condition translates into 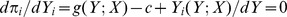. At the same time, for the total return 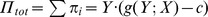, the maximum is achieved at 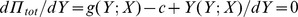. Noting that 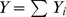, it is easy to see then that 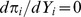 necessarily implies 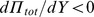 if 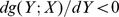, in other words, the Nash equilibrium corresponds to the players’ configuration where the returns are degrading, that is, the resource is overexploited.

We point out that the open market model described above is identical in its mathematical structure to the above tragedy of the commons. Specifically, the producers’ gains are defined by the same Eq. (1) and the market returns 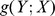 are likewise a decreasing function of *Y*. Then, similarly to the classical tragedy of the commons, it can be shown that in the Nash equilibrium of this model as well necessarily 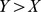, that is, the commodity is overproduced and the market is oversaturated. Intuitively, this result can be understood from the fact that the collectively “optimal” configuration, in which the supply and the demand meet, that is,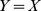, is unstable to unilateral increases in the production 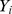 by any one of the producers, which allow that producer to increase her returns due to an associated increase in the market share 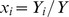. Of course, such an increase comes at the cost of the market shares and the profits of all the other producers. As a result of that, merely to maintain a parity in the market, all of the producers are subsequently led to increase their production outputs beyond the optimal point 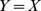, in order to counteract potential increases by their competitors. As a result, 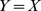 stops being an equilibrium point of the system and overproduction naturally develops as an outcome of such producers’ competitive behavior.

### Oscillatory Patterns in Open Market Economies

**Figure 2:**
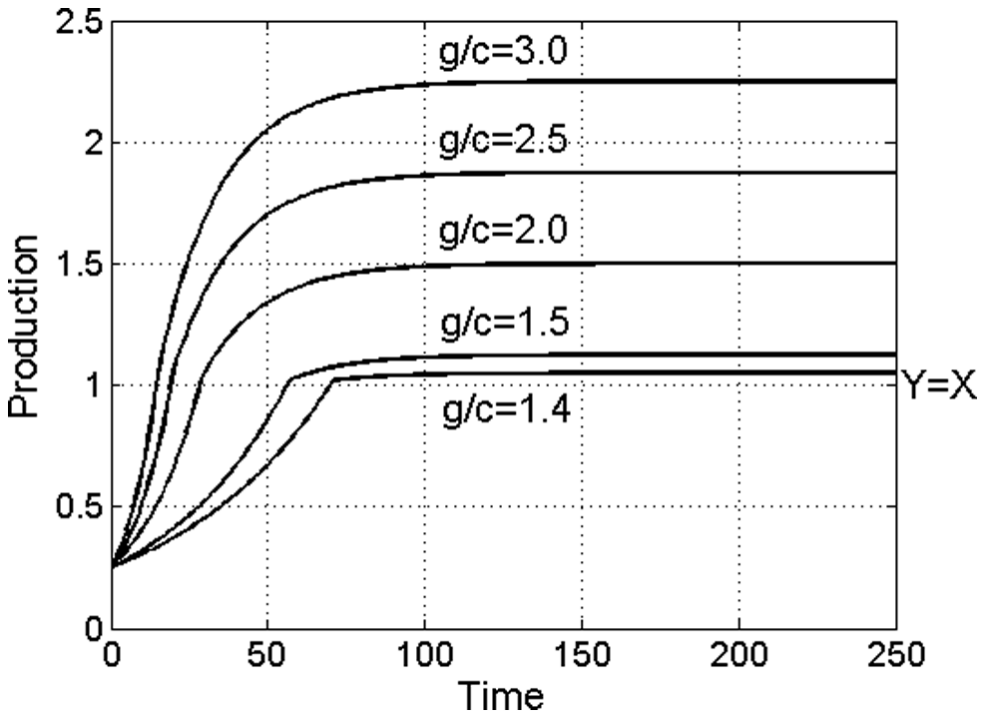
The market share competition of producers in a competitive open market economy should always result in overproduction of the commodity and oversaturation of the market, as shown in these market model dynamics. Overproduction by itself, however, does not necessarily trigger an economic recession, as the model production outputs observed here approach equilibrium point monotonically. In the graph, “*g*/*c*” stands for the profit margin used in each model and “*Y* = *X*” corresponds to the classical equilibrium point of equal supply *Y* and demand *X*.

Although overproduction crises are commonly stated as the leading cause of economic recessions [1], [14], [21], [23], in here we do not observe that the overproduction by itself necessarily causes a recession. In fact, in a dynamical simulation of the model (1) we observe that the model outputs converge to equilibrium monotonically and no recession occurs, Fig. 2. In Fig. 2 we graph a solution of the model (1) for different values of the return function *g*. Although the situation of overproduction indeed develops quite rapidly in these settings, the model does not exhibit subsequent crises and instead settles on an equilibrium monotonically. We, therefore, are led to conclude that overproduction and loss of profitability by businesses by itself is not sufficient for economic crises. If a recession is to emerge, a different mechanism is required to trigger drop in production.

We find such a mechanism by observing that allowing overproduced commodities to simply accumulate on the market over extended periods of time suffices to trigger oscillatory patterns in production outputs. More specifically, we describe the dynamic behavior of the producers in an open market by following relationships,
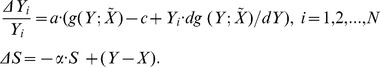
(2)


Here, the adjustments in the producers’ outputs 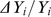 are driven by the expected profit 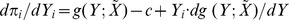, but the market size is taken in the form 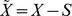, where *S* is the commodity that had been overproduced and is currently remaining on the market. The latter reflects the fact that previously produced and now persisting on the market commodity depresses the demand and the market for the new produce. The second equation describes the commodity’s accumulation on the market with the 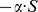 term modeling the commodity’s persistence on the market, whereas *α* represents the fraction of the overstock commodity lost naturally over one period of time.

**Figure 3:**
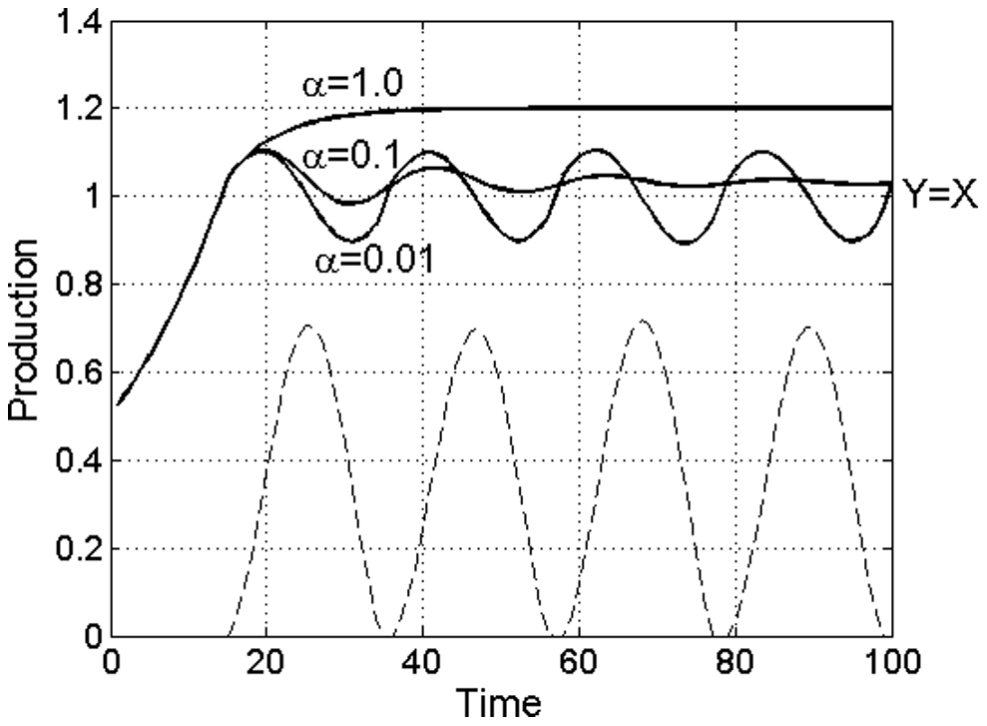
Cyclic recessions in model production outputs develop if overproduced commodity is allowed to accumulate on the market. The recessions, therefore, are triggered by the ability of overproduced commodity to accumulate on the market, that is, the model cycles develop in the markets of durable goods (small α) but not in nondurable goods (large α), similar to the business cycles in real economies, Fig. 1A. The dashed line shows the co-evolution of the commodity’s inventories during the cycle. Note the co-cyclic pattern similar to that observed in real economies, Fig. 1B. The simulation parameters: coefficient *a* = 1/20, profit margin *g/c* = 2, market size *X* = 1, the number of independent producers *N* = 4, the commodity’s persistence constants *α* = 0.01, 0.1 and 1.

To inspect the possible solutions of the model (2), we consider a simple instance of the model (2) in which the commodity’s price is assumed to be constant *g*. (Note that even in that case the return function 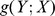 is not constant because in the market oversaturation regime, 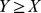, the amount of sold commodity saturates at *X* and the return per *Y* effectively drops as 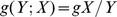). In that case, Eqs. (2) describe a linear dynamical system controlled by the following characteristic equation,
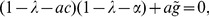
(3)where 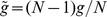. Depending on the value of the persistence constant *α*, therefore, Eq.(3) allows three different types of solutions. For large 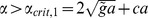, both roots λ_1,2_ of Eq. (3) are real and smaller than one, and the corresponding solutions of Eqs. (2) are non-periodic, α = 1 in Fig. 3. For small 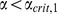, the roots become complex and the dynamical system (2) becomes periodic. Depending on the magnitude of λ_1,2_, however, one of two cases can realize here. For 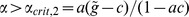, |λ_1,2_| are smaller than one and the dynamics is damped oscillations, α = 0.1 in Fig. 3. For 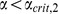, |λ_1,2_| are greater than one and the dynamical system (2) becomes unstable. The consequences of this instability are two-fold. Firstly, the corresponding market model ceases to have a stable equilibrium, that is, the cycles develop from any however small deviations from the exact equilibrium. Secondly, the oscillations become nonlinear – the excess commodity *S* is always reduced to zero at some point during the cycle and the cycle becomes self-sustained and non-decaying, α = 0.01 in Fig. 3. The cycle additionally becomes chaotic, as can be observed in the respective *Y*–*S* phase space trajectories of this dynamical system.

Depending on the persistence constant *α*, therefore, we observe three possible behaviors of the model (2). For large *α*, the production outputs approach the Nash equilibrium monotonically and no oscillations develop. In this case, the commodity does not accumulate on the market fast enough to trigger a recession and equilibrium is achieved directly. For *α* below a certain threshold, however, damped oscillations begin to develop, and yet for smaller *α* the model becomes unstable. In that latter case, persistent cyclic instabilities emerge from any however small deviations from exact equilibrium and develop into a self-sustained business cycle.

The situation of large α (low commodity persistency), evidently, can be taken to correspond to the situation with nondurable goods and services, while the case of small α (high commodity persistency) would correspond to the situation with durable goods and construction. Remarkably, these features of the model emerge also as a well-known property of real business cycles [21], [48], which are known to affect primarily durable goods manufacturing and construction, while leaving nondurable goods and services unaffected, Fig. 1A. Second striking feature of the model (2) is the co-cyclic behavior of the inventories, with excess accumulation of the inventories immediately prior to and drop during the recession part of the cycles, also well known for real business cycles, Fig. 1B.

## Discussion

In this work, we elucidate the development of cyclic instabilities in a fundamental economic model of an ideal single commodity open market with several producers. We observe that the root cause of these instabilities is a systemic overproduction caused by the competition of rational producers for market shares, followed by market depression due to an accumulation on the market of overproduced durable commodities. The possibility and the severity of such model cycles is found to be directly related to the ability of the commodities to accumulate on the market, that is, the cycles are observed for durable goods but not for non-durable goods or services. This feature of the model’s cycles is an otherwise well-known property of real business cycles [21]. The cycles are observed also to be associated with specific co-cyclic patterns in commodity inventories, with excess accumulation of inventories prior to and drop during the recession segment of the cycle, which is also a known property of real business cycles [48].

The emergence of cyclic instabilities with the key signatures of real business cycles in the above model is extremely striking. Single commodity market is one of the simplest and the most fundamental models in economics. Furthermore, we had to make no assumptions or artificial adjustments in order to observe the development of the cycles – the oscillations developed naturally from the fundamental properties of the model itself, namely, the strategic competition of the producers for market shares and the depressing effect of durable goods overstocks on the sales of the new produce.

As such, the described model invoked only pure market-driving forces, in the form of the strategic desire of market agents to maximize their profits and stocks-overstocks dynamics. It is clear, therefore, that the model’s behavior can change substantially in the presence of any additional regulatory mechanisms. In particular, the regulatory mechanisms affecting the types of behaviors touched upon in this paper can be expected to most significantly affect the persistence of business cycles. Such regulatory mechanisms, for instance, might include incentives for durable goods manufacturers that discourage them from attempting market share expansions in already saturated markets, or incentives for businesses aimed at discarding durable overproduced stocks at higher rates. Of course, any such regulatory options bring with them an entire array of complex technical, legal, social, and economic issues that cannot be possibly comprehensively examined in this work and shall warrant thorough investigation.

While one cannot expect the long-standing problem of business cycles to be resolved with any simple model of two variables such as described here, the simple findings presented in this work offer new insights into the long-standing issue of business cycles as a systemic property of efficient market systems emerging directly from free market competition itself and, therefore, intrinsic to open markets at a very fundamental level.
